# Large-area sensors using Cd(Zn)O plasmonic nanoparticles for surface-enhanced infrared absorption

**DOI:** 10.1515/nanoph-2025-0020

**Published:** 2025-05-26

**Authors:** Pablo Ibañez-Romero, Eduardo Martínez Castellano, Javier Yeste, Fernando Gonzalez-Posada, Thierry Taliercio, Vicente Muñoz-Sanjosé, Miguel Montes Bajo, Adrian Hierro

**Affiliations:** 16771 ISOM, Universidad Politécnica de Madrid, Madrid, Spain; Departament de Física Aplicada i Electromagnetisme, Universitat de València, Burjassot, Spain; Univ. Montpellier, IES. UMR 5214, F-34000, Montpellier, France

**Keywords:** CdO, Cd(Zn)O, SEIRA, localized surface plasmon, plasmonics, infrared

## Abstract

Surface-enhanced infrared absorption (SEIRA) spectroscopy holds significant promise for chemical detection as it enables direct identification of distinct vibrational fingerprints of molecules. Traditionally, SEIRA has been exploited through the use of tailored metallic nanoantennas, which are burdened by high losses in the mid infrared and costly nanofabrication techniques. In this work, we introduce an alternative, simpler approach using self-assembled Cd(Zn)O nanoparticles as a SEIRA platform. We demonstrate enhancements of the vibrational absorption of several molecular bonds in polymethyl methacrylate and vanillin up to a factor of 4.3. Such enhancements scale linearly with the surface density of nanoparticles, which can be controlled through the growth conditions. Thanks to the tunability of our platform, we report extended sensing capabilities to high energies in the mid-IR, reaching as high as 3,800 cm^−1^. All in all, we present a proof of concept for large-area, simple preparation, ready-to-sense surfaces that has the potential to be scaled up to become a prominent technology for mid-IR molecule sensing.

## 1 Introduction

Surface-enhanced infrared absorption (SEIRA) has become a popular method for sensing and quantifying very small quantities of organic molecules [1], [2]. Since its discovery in the eighties when it was first observed on rough metallic surfaces [3], there has been a wide specialization of the technique and it has demonstrated its worth in distinct fields such as electrochemistry, sensing, and safety [4], [5], [6]. SEIRA is enabled by the resonant coupling between a wideband plasmonic mode upheld by a nanostructure and a narrow resonance of an analyte for detection purposes. In this technique, the weak molecular absorption is not directly measured. Instead, its effect on the plasmonic spectrum is observed. The coupled harmonic oscillator model effectively represents the Fano-like shape of the SEIRA signal. This model includes the “bright” mode of the plasmonic resonator, which strongly interacts with far-field radiation, and the “dark” mode of the molecular vibration, which interacts weakly. Both modes exchange energy through a coupling factor increasing the extinction cross section [7]. Thus, the sensitivity is greatly enhanced when compared to conventional IR absorption techniques [8].

The mid infrared (Mid-IR) region of the electromagnetic spectrum holds significant potential for sensing applications [9], [10]. Specifically, between 1,500 and 3,600 cm^−1^, the so-called molecular fingerprint region [11] can be found, where most elemental bond stretching molecular resonances occur. It is the case of bonds like C–H, C=O, or O–H [12], which are present in inorganic compounds relevant in the detection of fire, greenhouse gases, and harmful gases [13]. Thus, big efforts are being undertaken by the scientific community to create the perfect platform to achieve efficient sensing in this range [14].

Traditionally, metallic nanostructures have been exploited for SEIRA spectroscopy [13], [15], [16], [17]. Particularly, noble metals such as gold and silver have been the natural choice for plasmonic devices due to their chemical stability. These materials are very efficient for sensing purposes in the visible and near infrared; however, when working in the mid-IR, they suffer great losses [18], [19], creating the opportunity for other novel materials to thrive in this range of the spectrum [20].

Highly doped semiconductors have been proposed as the perfect candidates for such job [21], [22], [23], [24]. They benefit from plasma frequencies (*ω*_p_) in the mid-IR region paired with low losses (*γ*), which allow for plasmonic resonances in the high energy region of the mid-IR (3–10 μm, or 3,333-1,000 cm^−1^) [25]. Remarkably, their plasma frequency can be tuned just by adjusting the doping level, which allows for accurate tailoring of the sensing devices, something unachievable with noble metals [26].

Among doped semiconductors, CdO stands out as the plasmonic material with the highest figure of merit (*ω*_p_/*γ* ratio) in the mid-IR, as highlighted by Caldwell et al. [27], especially for localized surface plasmons (LSPs). Notably, CdO features a very high plasma frequency of 3,100 cm^−1^ and optical losses of 550 cm^−1^ [28], or even lower [27]. Such properties can be further enhanced by alloying the material with Zn, thereby creating a ternary compound, Cd(Zn)O, which exhibits plasma frequencies exceeding 4,000 cm^−1^ and losses around 500 cm^−1^ [29]. Indeed, there are already a large variety of plasmonic effects and applications where CdO and Cd(Zn)O have been applied [24], [27], [28], [29], [30], [31], [32], [33], [34].

The main trend on SEIRA using highly doped semiconductors is to utilize highly engineered nanostructures fabricated using electron beam lithography (EBL) [19], [35], [36], [37], [38], [39]. Given the high control of the geometry in this technique, a wide variety of nanoantennas can be designed that allow to control the LSP frequency and the total enhancement of the electric field. Typical configurations include longitudinal rods [40], bowtie antennas [41], honeycomb antennas [42], etc. These architectures take advantage of the lightning rod and gap effect, where the electric field is highly concentrated around the edges and gaps between the nanostructures. It is in these areas, called hotspots, that the SEIRA enhancement naturally occurs. These studies, however, have an intrinsic economic and time cost derived from the use of EBL [43]. Consequently, such approaches face important adversities for scaling the designs to ready-to-use sensing devices for commercial applications. As a result, novel paths for SEIRA sensing with semiconductors are being developed that avoid the use of EBL. Some of them include techniques with potential for mass production such as atomic layer deposition [44], [45], nanosphere lithography [46], [47], or molecular self-assembly [48], [49]. This variety of solutions portrays the prosperity of this field, pushing forward commercial SEIRA-based sensors.

In this work, we propose a lithography-free, highly sensitive approach for SEIRA sensing in the 3–6 μm range of the mid-IR. Self-assembled Cd(Zn)O nanoparticles (NPs) with $0\leq \left[\text{Zn}\right]\leq 20\;$ % are grown by metal organic chemical vapor deposition (MOCVD) on highly mismatched semi-insulating GaAs substrates, providing a simple cost-efficient approach. The plasmonic response of the nanoparticles reaches as far into the mid-IR as 4,000 cm^−1^, proving to be an outstanding framework for molecular fingerprinting of various bonds found in most organic compounds. Most importantly, the NPs SEIRA performance yields a signal enhancement up to a factor of 3 for thin layers of PMMA (15 nm) and above 4 for a highly diluted solution of vanillin.

## 2 Results and discussion

In this work, two series of samples are considered, a first series (A) with increasing Zn alloying levels to identify the case with the best plasmonic behavior, and a second series (B) with different NP density to study the influence of the surface coverage on the SEIRA performance. Table S1 summarizes the samples’ characteristics and growth parameters.

**Figure 1:**
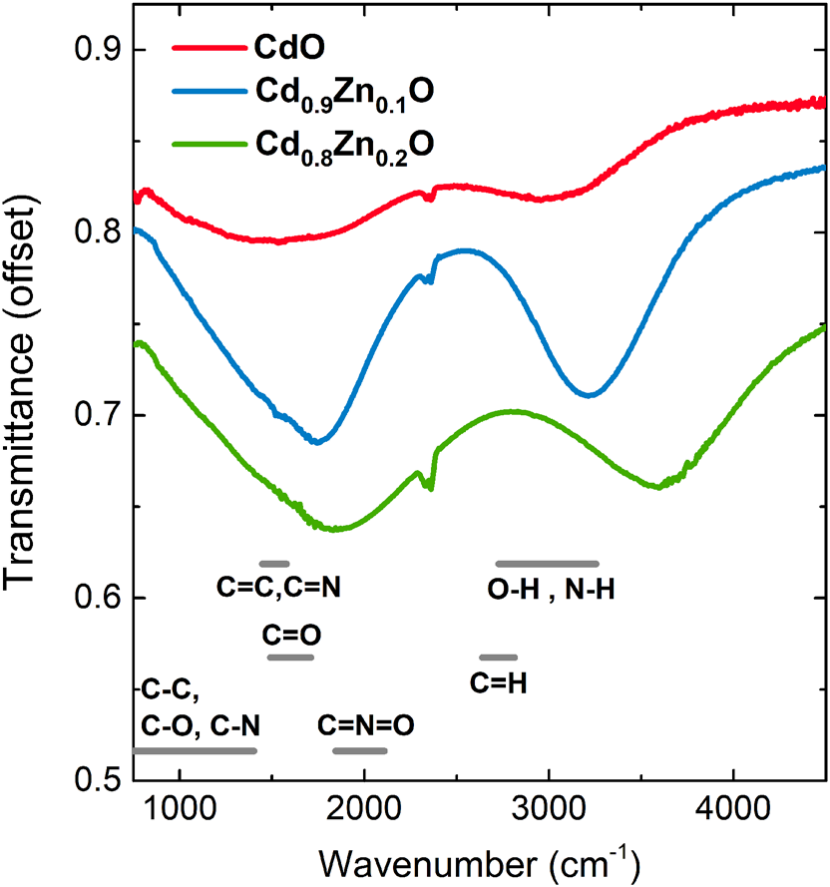
Measured transmittance spectra of three Cd(Zn)O NP samples with different Zn alloy compositions at 45° incidence. The most significant absorption bands from molecular bonds are also shown.

Figure 1 shows transmittance spectra for samples A1 (0 % Zn), A2 (10 % Zn), and A3 (20 % Zn). The observed plasmonic response of the NPs consists of two distinct LSPs modes. The lower energy mode corresponds to plasma oscillations parallel to the substrate, while the higher energy mode corresponds to out-of-plane oscillations, as discussed in Ref [33]. It is apparent how both LSPs are deeper and narrower for sample A2. In fact, if the quality of the resonances is measured as the ratio of the frequency (*ω*_LSP_) to the width of the LSP (*γ*_LSP_), the sample with 10 % Zn (A2) presents the best figure or merit for both LSPs, as shown in Figure S4.2 of the Supporting information. Therefore, the rest of the study is carried out using NPs with 10 % Zn, which yield figures of merit of 1.95 for the low energy mode and 4.8 for the high energy mode. These values are comparable to those previously reported in the literature for In-doped CdO nanostructures [34].

The evolution of the LSPs quality with Zn content matches the evolution of the ratio of plasma frequency to losses with Zn content in thin Cd(Zn)O films reported elsewhere [29], which features a maximum at around 10 % Zn, and can therefore be attributed to the same origin. Nevertheless, the potential impact of NP morphology and distribution is also next considered. Figure S1 shows that samples with 10 and 20 % Zn are similar to each other in terms of NP distribution, whereas the sample with 0 % Zn shows smaller and more densely packed NPs, about to coalesce on some places. According to the simulations shown in Figure S2.1, coalescence results in transmittance spectra getting gradually closer to that of a thin film. This might also have an impact on the low quality of the LSP resonances shown in Figure 1 for sample A1.

The width of the LSP modes in Figure 1 can be attributed to two effects: homogeneous broadening, given solely by the material properties of the NPs, which is further discussed in Section S2 of the Supplementary material, and inhomogeneous broadening, which results from the distribution of sizes and locations of the NPs and will be addressed later. Both effects result in broad LSPs, which can cover a wide range of frequencies, from 1,000 cm^−1^ all the way to 3,500 cm^−1^. This feature of the NP’s behavior allows broadband sensing in the fingerprint region of the mid-IR, where most elemental bond stretching resonances in organic molecules fall (Figure 1). Incidentally, the Zn content of the NPs shifts the LSPs due to the changes in the plasma frequency of the alloy [29], but this is a small effect compared to the width of the peaks, experimentally masking any detuning effects [50]. It is not the intention in this study to tune the plasmonic resonances to a certain frequency like in previous studies [51], [52], [53], but rather to benchmark the best conditions for developing a sensing platform based on Cd(Zn)O NPs.

To evaluate the dependence of the NP size and surface coverage on the growth parameters, a series of four samples were synthesized with different growth parameters (see Methods) but keeping the Zn content at 10 %. Their growth conditions are collected in Table S1, under series B. The NPs in this study follow a self-assembly process that results in a distribution of particles with different sizes and surface coverages. In order to statistically analyze these distributions, a combination of atomic force microscopy (AFM) and scanning electron microscopy (SEM) images were used to provide a precise understanding of the microstructure of the NPs.

**Figure 2:**
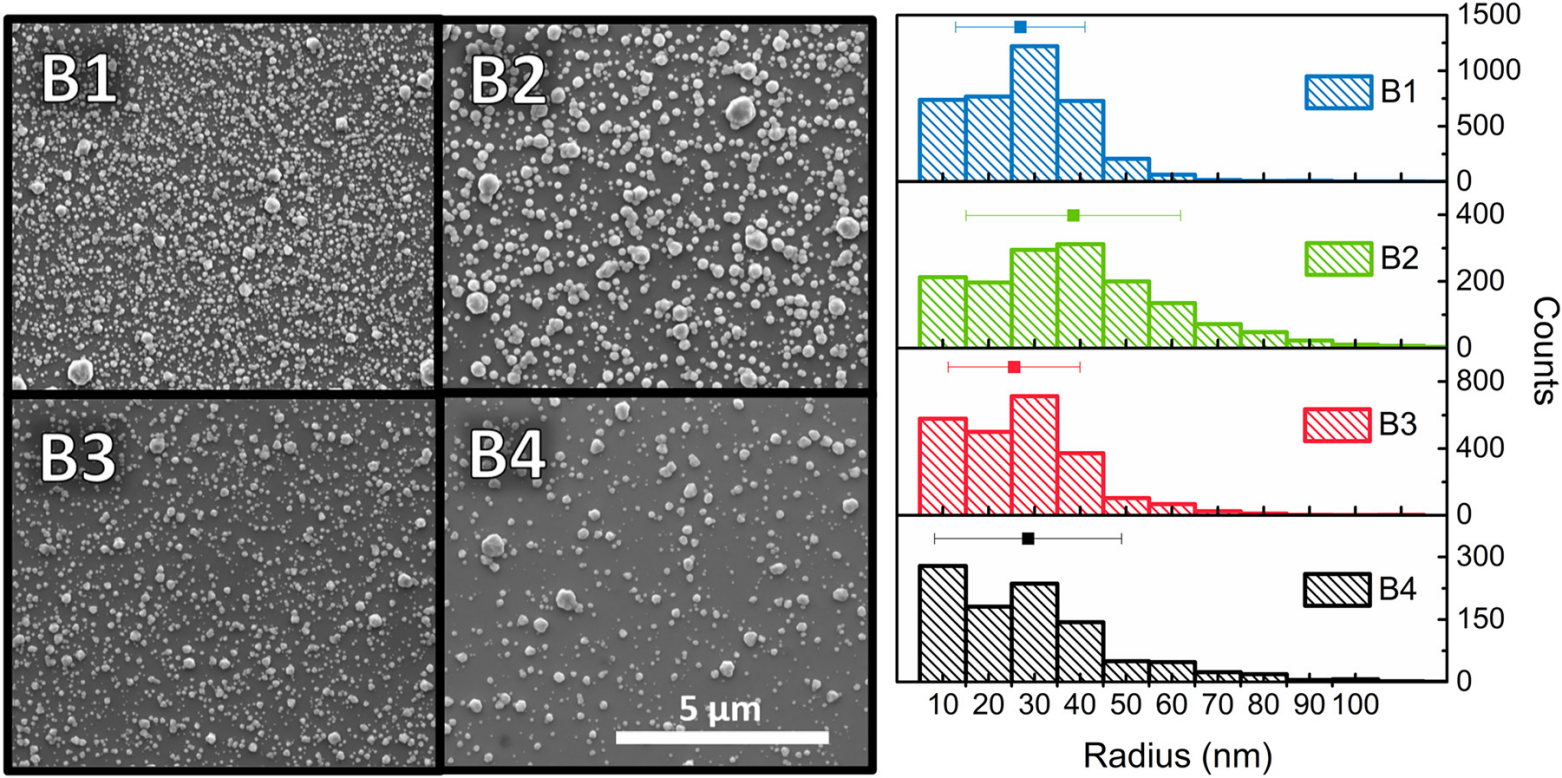
Scanning electron micrographs of the surface of samples B1, B2, B3, and B4 with different surface coverages of Cd_0.9_Zn_0.1_O nanoparticles. The histograms portray the size distribution of the NPs, together with the average radius and its standard deviation.

Figure 2 shows a representative SEM image of the surface of the samples, along with a histogram of the size distribution of the NPs for every sample. The main statistical characteristics of the NP distribution are summarized in Table 1, and their reproducibility is confirmed by previous reports from the authors [54]. As can be seen in this Table, there is a wide range in surface coverages, extending from 38 % in sample B1, to 12 % in sample B4. As will be discussed later, this wide range of surface coverages serves as a route to control the intensity of the SEIRA signal. Furthermore, as shown in Figure 2, the size of the NPs follows a distribution with a mean radius around 30 nm, and typical standard deviations between 14 and 23 nm. However, as a result of their deeply subwavelength size, the sizes of the NPs have a very small impact in SEIRA sensing. We show in Figure S3.1 that varying the NP size from 10 to 50 nm yields minimum changes in the LSP modes.

**Table 1:** Statistical parameters of the NP distribution extracted from the histograms in Figure 2.

Sample	Zn content (%)	Surface coverage (%)	Number of NPs in 25 µm^2^	Average NP radius (nm)	NP radius standard deviation (nm)
B1	10	38	3,772	27	14
B2	10	30	1,526	38	23
B3	10	22	2,375	26	14
B4	10	12	999	29	20

**Figure 3:**
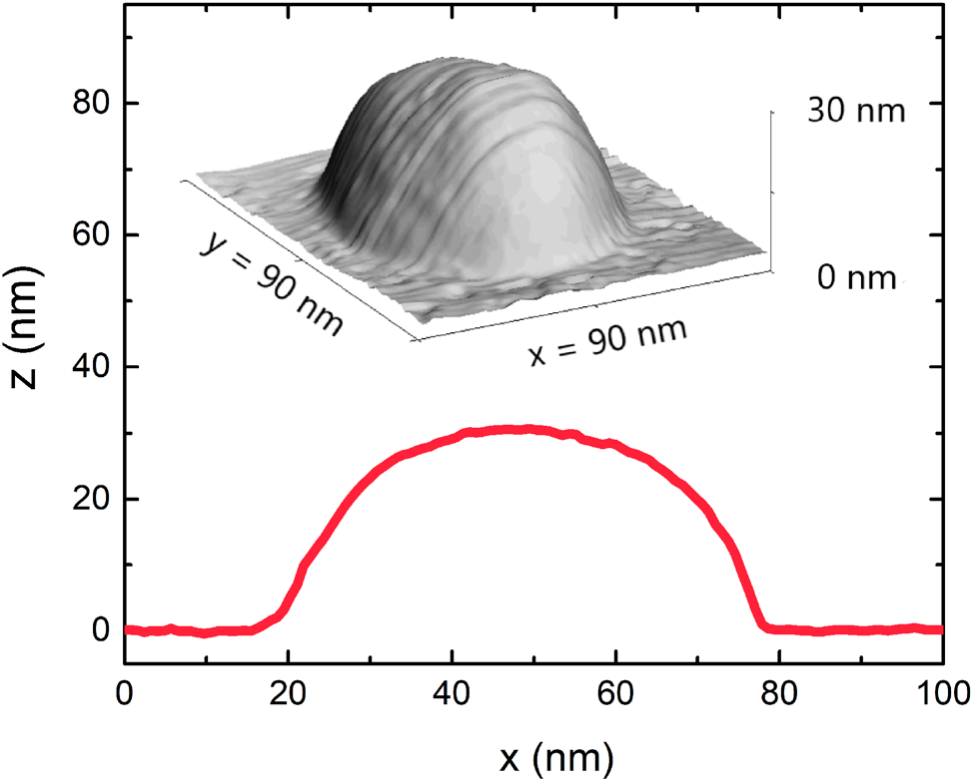
Top: AFM image of a single NP. Bottom: Cross-sectional profile of the NP at the apex.

Regarding the geometry of the NPs, a 3D AFM measurement of a representative NP is shown in Figure 3. The shape resembles an almost perfect hemisphere as proven by the lateral cross section shown in the figure. The geometry of this NP is the main input of the finite element model (FEM) used for the simulations displayed later in this work, whose validity is shown in Figure S3.1 revealing excellent agreement between the model and the empirical results. The only noticeable discrepancy is a slight reduction in the LSP intensity in the simulated spectra, which is likely attributable to interparticle coupling effects present in the experimental samples. Due to the random spatial distribution of the NPs on the substrate, a fraction of them are positioned in close proximity to each other, leading to near-field coupling and enhanced extinction, as illustrated in Figure S2.2. This coupling is not incorporated into the FEM model, potentially accounting for the reduced depth of the transmittance minima observed in the simulations (Figure S3.1). The statistical analysis of the NPs’ shape and distribution, and the good agreement between modeled and measured reflectance spectra (Figure S4.1), justifies modeling them as hemispherical NPs with 60 nm in diameter. Further discussion on the finite element model’s (FEM) geometry and validity is provided in the Supporting information (Sections S3 and S4).

**Figure 4:**
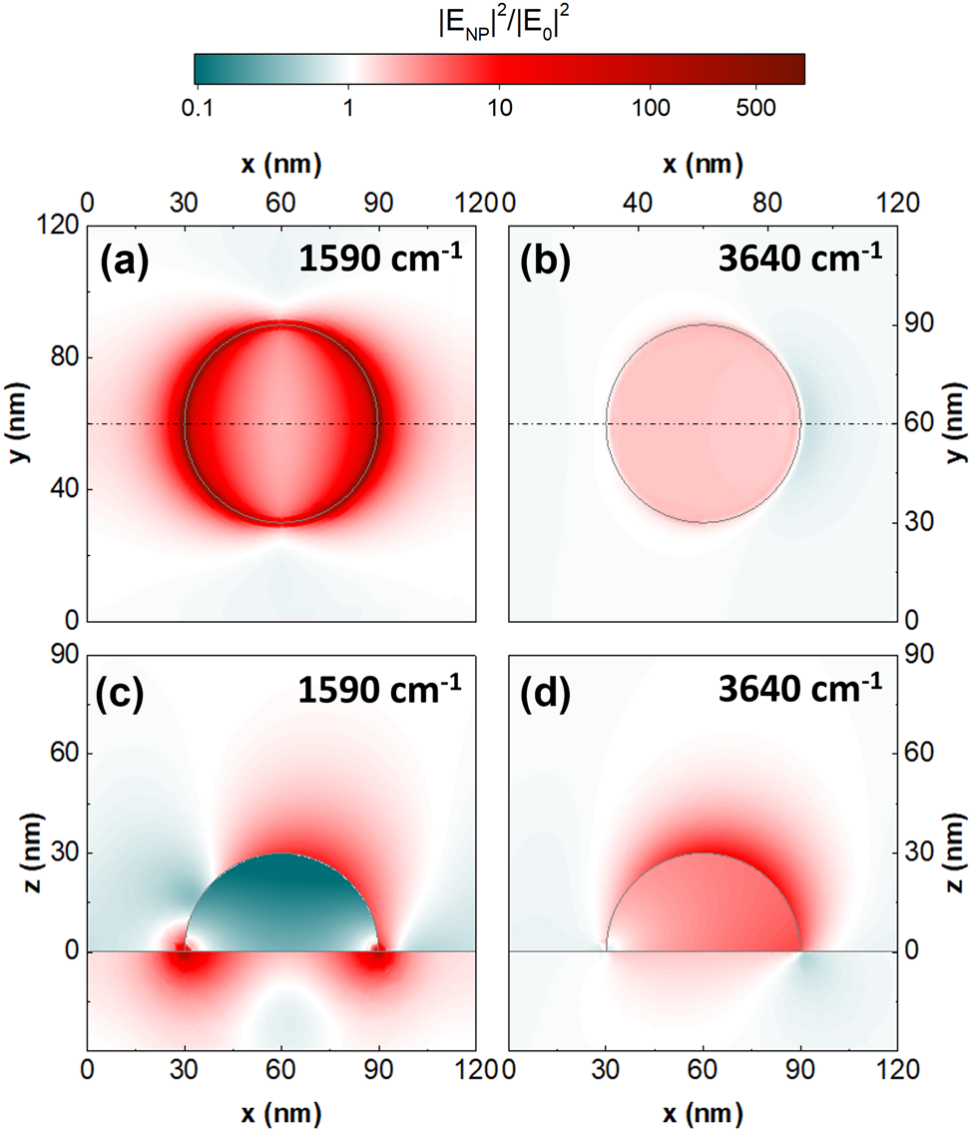
Near-field enhancement on NPs. (a) and (b) Show the normalized field enhancement for the low and high energy LSP modes for the cross section of the NPs at *z* = 0 nm, where they meet the substrate; (c) and (d) show the normalized field enhancement for the low and high energy LSP modes for the cross section of the NPs at *y* = 60 nm, i.e., through the center of the NP marked by the black dotted line. The angle of incidence is 45° in all cases. The white color represents the areas where there is no enhancement when compared to a model without the NP.

Lastly, it is important to analyze the NP plasmonic modes in the near field to better understand how they behave, and how they can be used for SEIRA sensing. Figure 4 shows the FEM model results for the light intensity enhancement in and around Cd_0.9_Zn_0.1_O NPs, which is given by |E_NP_|^2^/|E_0_|^2^. The model was calculated at 45° incidence for the frequencies where the NPs undergo their respective plasmonic modes. The two top figures (a and b) show the cross section of the NPs at *Z* = 0 nm, where they meet the substrate, and the two bottom (c and d) ones its cross section at *Y* = 60 nm, i.e., through the center of the NP. At 45°, both the high and low energy modes can be exited, as previously described [33].

For the low energy mode (1,590 cm^−1^), the electric field is largely enhanced at the points where the NPs meet the substrate, reaching high enhancement values of up to 150 (Figure 4a). In the cross section presented in Figure 4c, it is clear that the field enhancement is below one inside the NP, which implies that it is smaller than it would be if the nanostructure was not there. This is due to the fact that 1,590 cm^−1^ is well below the plasma frequency of Cd_0.9_Zn_0.1_O, and so the material’s behavior is similar to that of a metal, preventing the electromagnetic wave from penetrating the NP. On the other hand, the high energy mode is located at 3,640 cm^−1^ (Figure 4b), close to the bulk plasma frequency of the material, allowing the wave to penetrate into the NP as we see in Figure 4d. Most importantly, the enhancement for this mode is considerably smaller than that of the low energy mode, only achieving a maximum value of 4 around the apex of the NP. This will become relevant when analyzing how the system performs in sensing.

### 2.1 SEIRA sensing

The analysis of SEIRA with NPs was initially approached by a far field study using the techniques described in the methods section. Sample B1, which has the largest surface coverage, was chosen to exemplify the detailed analysis of the SEIRA performance carried out in all of the samples of the series since it is the one with the best performance. The results for such sample are presented below, and in a latter section, those of the whole series.

**Figure 5:**
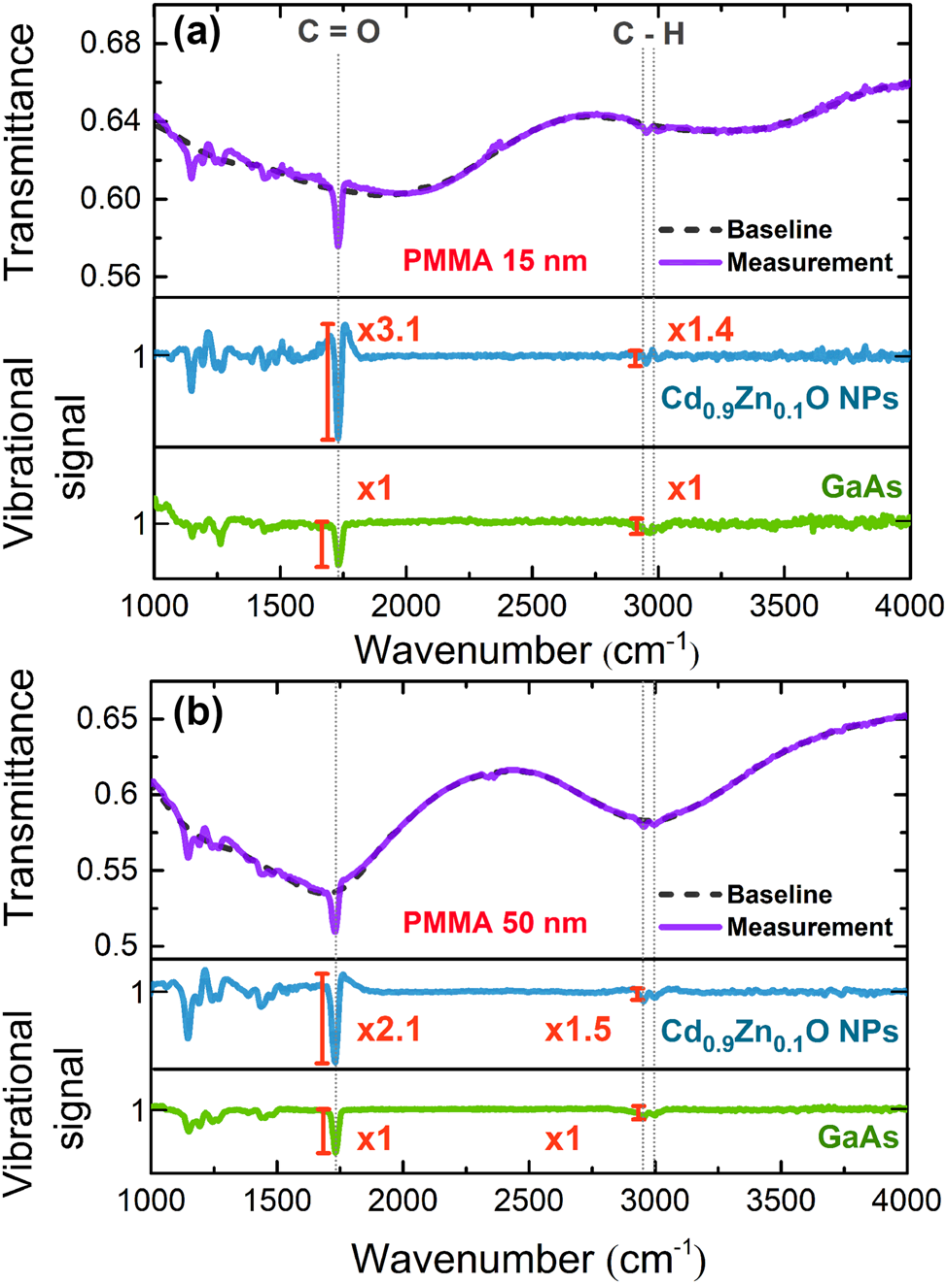
Transmittance spectra (purple) of Cd_0.9_Zn_0.1_O NPs with a 15 nm (a) and 50 nm-thick (b) layer of PMMA spinned on top of the sample. In both (a) and (b), the purple line corresponds to the measurement, the dotted black line corresponds to the baseline obtained by using Eilers method (see Methods), and the blue line to the vibrational signals obtained upon subtraction of the baseline from the measurement. The green curve shows the signal from PMMA deposited on a bare GaAs substrate and serves as a reference. Hence, the enhancement in the absorption lines is obtained by normalizing the amplified signal by that from the reference.

To better understand the mechanisms underlying the SEIRA enhancement, the transmittance spectra of sample B1 were obtained for two cases, with a 15 nm or a 50 nm-thick layer of PMMA deposited on top of it. Both spectra were analyzed as described in the methods sections, and the results are shown and compared with a reference sample of PMMA-coated GaAs substrate in Figure 5. In it, the asymmetric s-like shape of the Fano resonance can be seen, indicative of the resonant coupling of the absorption from the molecule and the LSP mode from the nanoparticles [40]. For the 15 nm-thick PMMA case, and using the coupling between the low energy LSP mode and the molecule bond, we report an absorption enhancement factor of 3.1 for the vibrational signal from the stretching mode of the C=O bond (1,732 cm^−1^) of PMMA. This magnitude of enhancement is comparable to previously reported values in both metals and semiconductors [13], [14], [15], [16]. Also note that by depositing PMMA on the surface of the sample, the permittivity of the environment that surrounds the nanoparticles is changed, producing a slight shift of the plasmonic resonances when compared to the spectra shown in Figure 1. Similarly, the molecules’ interaction with the substrate and the NPs directly influences their binding energy. As a result, the experimentally observed vibrational lines exhibit a slight infrared shift from those found in the literature.

**Figure 6:**
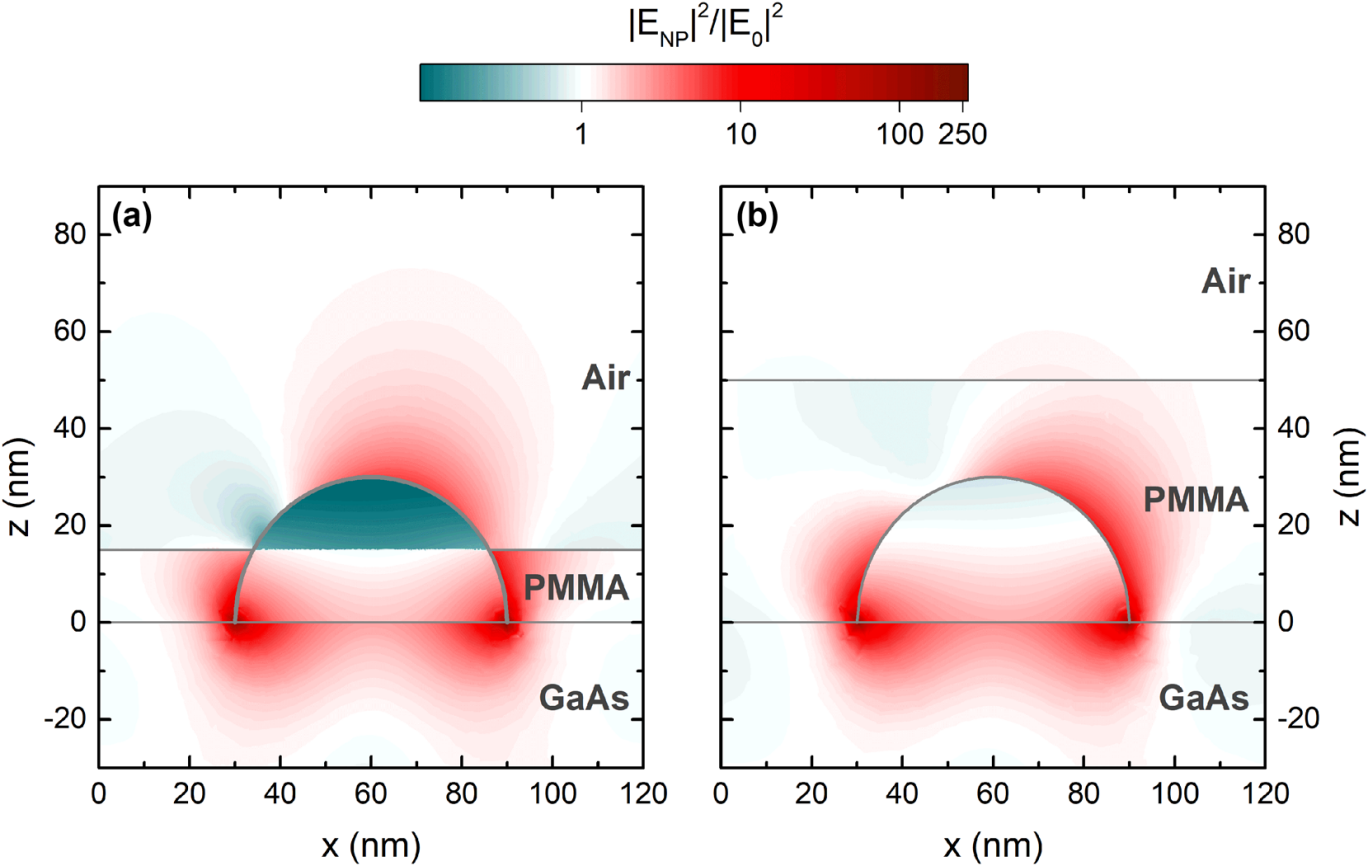
Cross-sectional normalized field enhancement at 1732 cm^−1^ for the low energy LSP mode of the NPs covered with a 15 nm (a) and 50 nm-thick (b) film of PMMA at an incidence angle of 45°. The white color represents the areas where there is no enhancement when compared to a model without the NP.

Figure 5 also shows the spectra for the case of a 50 nm-thick PMMA coating. In this case, the vibrational signal enhancement is a factor of ×2.1, considerably smaller than that of the 15 nm-thick film of PMMA. There are two possible explanations for this matter. The first one can be extracted from observing how SEIRA is originated in the near field, and the second one from studying the frequency detuning. To begin with, Figure 6 presents the near field enhancement (|E_NP_|^2^/|E_0_|^2^) for a canonical NP with both 15 and 50 nm PMMA coverages. As can be seen, the field is highly amplified around the vertices where the NP meets the substrate. It is around these areas, named hot spots, where PMMA experiences the largest absorption enhancement. Hence, it becomes clear from the results in Figure 6 that the ratio of total PMMA volume where there is high near field amplification is smaller in the thickest film. This explains well the smaller amplification experimentally observed in Figure 5 for the 50 nm-thick PMMA film.

In terms of frequency detuning, the low energy mode is at a higher energy than the molecular absorption of the C=O bond for the 15 nm case but resonant for the 50 nm case. As reported by Vogt et al. [50], the slight detuning of the molecule absorption line (*ω*_C=O_) from the plasmonic resonance frequency (*ω*_LE_) results in a higher vibrational signal. This results in the vibrational signal for the 15 nm PMMA layer being higher than that of the 50 nm one, as the frequency tuning ratio (*ω*_C=O_/*ω*_LE_) is around 0.87 compared to 1, respectively, as shown in Figure 5. Ultimately, both the influence of the field distribution with the PMMA thickness and the frequency detuning contribute to the vibrational signal being larger for the 15 nm PMMA film case.

The stretching of PMMA’s C–H bond has absorption frequencies at 2,956 and 3,001 cm^−1^ (2,947 and 2,986 cm^−1^ in the literature due to the aforementioned shift), which aligns perfectly with the frequency of the high energy LSP mode of the NPs. Thus, a coupling between the bond and the LSP mode is expected to occur, and indeed it is visible in Figure 5, yielding an enhancement factor of 1.5. However, the enhancement factor for the C–H bond coupled to the high energy LSP mode is clearly smaller than that of the C=O stretching bond coupled to the low energy LSP mode. As discussed earlier, this is likely a result of the much lower amplification of the electric field provided by the high energy LSP mode. Further, this amplification appears on the apex of the NP (see Figure 4), which could explain why with the thickest PMMA layer, covering entirely the NP, the resonance from the bond shows a slightly higher enhancement. Given that both the enhanced and unenhanced material in the 50 nm layer is bigger than in the 15 nm layer, it adds up to similar vibrational enhancement overall. In terms of detuning, the trend is the same as for the low energy mode, which could also explain the slight difference in vibrational signal; however, the change is too small (0.1) to reach further conclusions as both mechanisms contribute to the effect.

The geometry of our samples offers a radical departure from most recent studies regarding semiconductor nanoantennas for sensing applications. As compared to evenly spaced devices that use electron beam lithography [13], [14], [15], [16], Cd(Zn)O self-assembled NPs provide a stochastic approach that requires an alternative analysis. Nevertheless, we first need to understand how SEIRA is usually quantified. The figure of merit typically used to quantify SEIRA sensing is the Enhancement Factor (EF), which is defined as [2]:

$$EF=\frac{S_{NP}}{S_{0}}\times \frac{A_{0}}{A_{NP}}$$

where *S*_NP_ represents the enhanced signal strength, while *S*_0_ denotes the unenhanced signal strength. *A*_NP_ and *A*_0_ both represent areas covered with molecules, the former accounting for those in contact with the hotspots, and the latter for the entire surface of the unit cell. Notably, the active area *A*_NP_ is not precisely defined and requires careful consideration, being commonly assessed indirectly via electromagnetic simulations [2]. Since we have a thin analyte film covering the whole surface of the sample, it is fair to assume all the hotspots are in contact with analyte molecules. The ambiguity would be in the condition to consider what is a hotspot (i.e., the threshold field amplification), which is arbitrary. This arbitrariness together with the possibility to modify the ratio *A*_0_/*A*_NP_ by changing the density of hotspots is what compels us to avoid using the EF.

Therefore, although the EF is established as the norm, we consider it is not the fairest metric for our samples due to the high density of hotspots. We believe that the vibrational signal is a better guiding parameter for these types of studies, as the widely accepted EF fails to provide a consistent approach. However, in order to benchmark our results with the previously reported bibliography, we must provide an estimate of such metrics.

**Figure 7:**
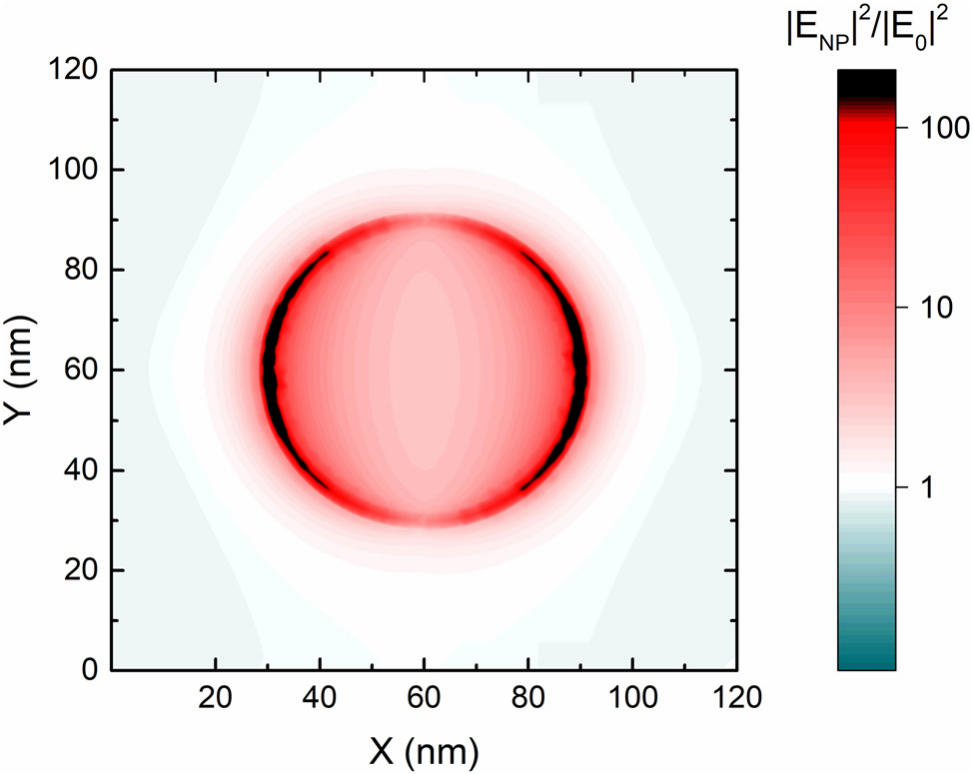
Plan view of the normalized field enhancement for the low energy LSP mode of the NPs at an angle of 45°. The black regions correspond to an enhancement of |E_NP_|^2^/|E_0_|^2^ > 150.

In our case, *A*_NP_ can be extracted from the plan view image of the NP present in Figure 7. The field is highly enhanced and concentrated in the areas that appear black in the colormap. Such areas correspond to a cutoff of |E_NP_|^2^/|E_0_|^2^ > 150. With the help of ImageJ, the total area in white (*A*_NP_) can be quantified, yielding a result of 94.5 nm^2^. On the other hand, the total area of the unit cell, *A*_0_, is computed just as the square of its side, resulting in a value of 1.44 × 10^4^ nm^2^. Thus, the ratio *A*_NP_/*A*_0_ yields a value of 153, which multiplied by the vibrational enhancement results in the EF. From our previous discussion, we have stablished that the maximum enhancement that we have observed is for the C=O stretching vibrational signal for the 15 nm-thick PMMA film. In this case, the *S*_NP_/*S*_0_ ratio equals 3.1, yielding an EF of 4.73 × 10^2^, a value comparable to the values reported by Huck et al. using metallic nanorods [2]. However, such EF value is 2 orders of magnitude below most current studies [40]. As an example, the study by V. Vogt, where nanoantennas arrays are processed through e-beam lithography, reports EFs up to 2.7 × 10^4^, while the maximum enhancement in the vibrational signal is 2.11 [50]. This means that even though their reported signal enhancement is 33 % smaller than the one that we report, their geometry allows an EF 20 times larger than ours. Other reports on semiconductor nanostructures [52], [55], [56], [57] show EFs in a broad range, but when it comes to vibrational enhancements, the reported values are found within the order of magnitude of the results from this article.

From the application standpoint, we believe that our approach offers realistic applicability. We argue that in the realm of commercial sensing devices, instead of devices where the enhancement per nanostructure is extremely high but the overall acquired signal is very low and costly to probe, it would be more beneficial to obtain a much larger signal by greatly increasing the number of nanostructures per unit area that provide SEIRA (i.e., increasing the ratio of SEIRA-active area to total area of the sample), as we propose. This large number of NPs not only yields a larger signal but also greatly increases the probability of the probed analyte to be found around one of them. Furthermore, as the Cd(Zn)O NPs are formed by a self-assembly process, the device fabrication would be faster and more cost-efficient than reported approaches that involve electron beam lithography. In fact, our proposed approach uses readily available substrates and allows to coat them with NPs yielding an area as large as 2 cm^2^. Such large coating area also facilitates the implementation of our transducers into commercial detectors, as the spot size of the illumination sources available is in the realm of the millimeters.

**Figure 8:**
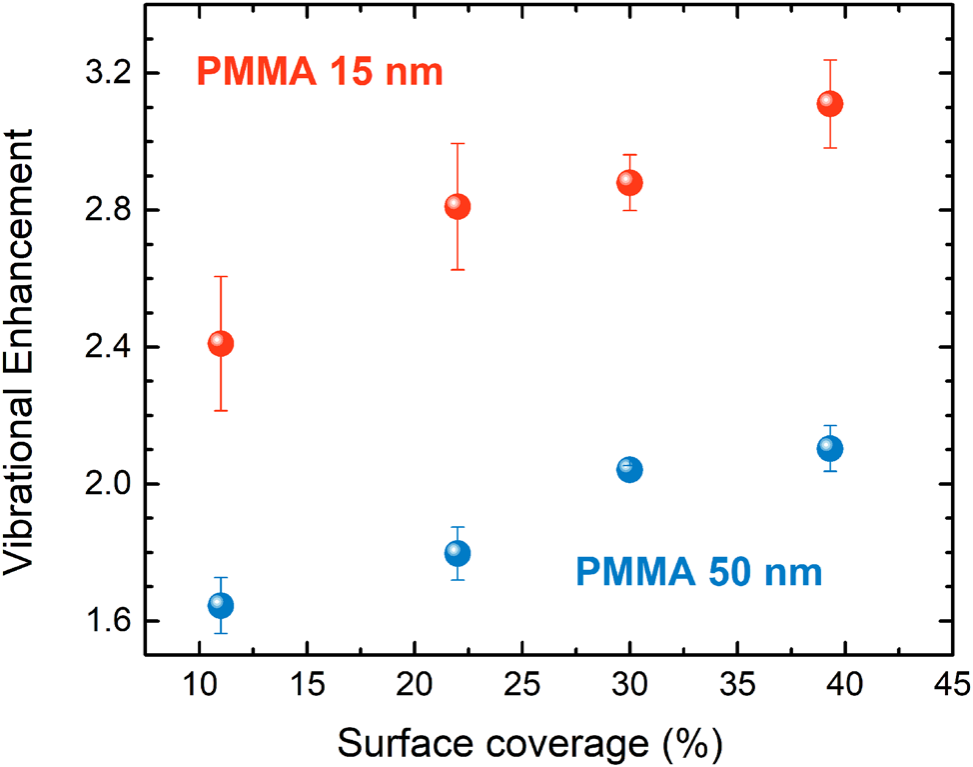
Dependence of the vibrational signal enhancement of the C=O stretching bond coupled to the low energy LSP mode, on the NP surface coverage for the 15 nm (red) and 50 nm-thick (blue) PMMA films. The error bars included in the graph account for the standard deviation from the mean, which is due to heterogeneities found in the samples as the experiments were performed on eight different spots on the surface of each sample.

As pointed out above, it is clear that the density of nanoparticles strongly influences the total enhancement of the vibrational signal. Thus, a study of the four samples with different surface coverages (micrographs in Figure 1) is due. The samples were processed, measured and analyzed as explained earlier, yielding the SEIRA signals that are shown in Figure 8, for both the thicker and the thinner PMMA coatings. The results, derived from the vibrational coupling between the low energy LSP mode and the C=O stretching bond of PMMA, show a clear linear increase of the vibrational enhancement with the surface coverage, thus confirming our hypothesis. This implies that, as long as the NPs do not coalesce, the increase in surface coverage through maximization of the number of NPs carries an increase of the effective number of hotspots where the absorption of the PMMA film is enhanced.

Lastly, a study of SEIRA was also conducted to test the efficiency of our device on sensing other molecular bonds. A useful compound to do so in the mid-IR is vanillin as it features several strong IR active vibrational lines of diverse functional groups. Within a range of less than 750 cm^−1^, we can find the bending of the CH_4_ and C–H bonds as well as the stretching of the C=O, C–C, C–O, and CH_3_ bonds, all of them spectrally located within the wide low energy LSP mode of the Cd_0.9_Zn_0.1_O NPs.

As specified in the Methods section, approximately 10 nmol of vanillin (molecular mass of 152.1 g/mol) are deposited uniformly on the entire 0.25 cm^2^ surface of the transducer. If we assume that the adsorption of vanillin onto GaAs follows a Langmuir isotherm model [58], then a monolayer coverage is achieved with noninteracting adsorbed molecules. This results in approximately 2 ng of vanillin within a 200 × 200 μm region, which is the area under illumination. An area equal to the unit cell described in the model, *A*_0_, contains 9 × 10^−5^ pg per NP, which means there are roughly 3 × 10^5^ molecules per NP. However, the vibrational enhancement is only given by the molecules that are primarily located in the intense near-field hotspots of the NP. As the effective area accessible to the analyte (*A*_NP_,) represents a 0.65 % of *A*_0_, we can conclude that approximately 2 × 10^4^ molecules are triggered per NP. This number is two orders of magnitude larger than the state of the art in SEIRA sensing [13].

**Figure 9:**
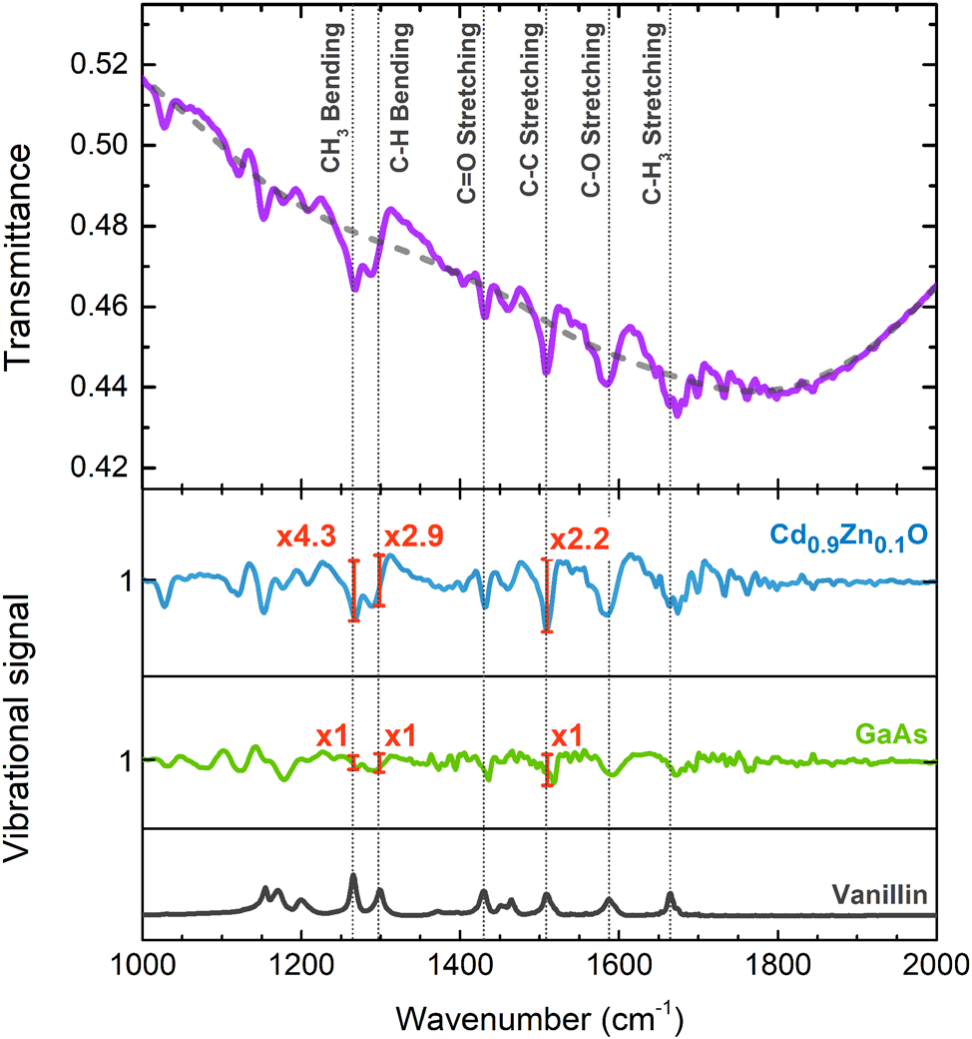
Transmittance and SEIRA vibrational enhancement for Cd_0.9_Zn_0.1_O NPs covered with vanillin. The purple line corresponds to the measurement, the dotted gray line corresponds to the baseline obtained by using Eilers method (see Methods), and the blue line to the vibrational signals obtained upon subtraction of the baseline from the measurement. The green curve shows the signal from vanillin deposited on a bare GaAs substrate and serves as a reference. Hence, the enhancement in the absorption lines is obtained by normalizing the amplified signal by that from the reference. In black, vanillin’s main absorption lines to serve as a guide.

The results of the study are presented in Figure 9. The black spectrum on the bottom graph serves as a guide to observe the resonance frequencies of the molecular bonds. A very large enhancement of the vibrational signal of most molecule resonances is found again, being the CH_3_ bending the largest one, with an enhancement factor of ×4.3. Other peaks, such as the C–H bending and the C–C stretching bonds, also show high levels of enhancements comparable to the ones discussed in the previous study for PMMA. Given such vibrational enhancement, the achieved EF is 1.5 × 10^3^, again a value in the order of magnitude of the current state of the art.

A question now prevails: why is the EF of vanillin larger than that of PMMA for the same surface architecture? Even though we do not have the means to realize an in-depth study on this issue, we propose the following hypothesis. When dissolved, vanillin is a small molecule, while PMMA is a polymer chain formed by the addition of sequential monomers, resulting in particles sizes that can range from hundreds of nanometers to hundreds of micrometers. This outstanding size difference ultimately means that the access of the molecules to the hot spots in the NPs is radically different between both compounds. On one hand, the small size of the vanillin molecules may allow them to adsorb easily anywhere on the surface, including the vertices where the NP meets the substrate and where the electric field is highly enhanced. On the other hand, the size of the PMMA chains could prevent them from fully covering the mentioned hotspot, thus showing a smaller coupling and ultimately a lower vibrational enhancement.

## 3 Conclusions

In this work, we demonstrate SEIRA with self-assembled Cd(Zn)O NPs. High vibrational enhancement values are revealed for several molecular bond resonances, tripling the absorption of the C=O stretching bond in PMMA and quadruplicating that of the CH_3_ bending bond in vanillin. Due to the exceptional properties of Cd(Zn)O as a plasmonic material, high energies of the mid-IR spectrum can be sensed using this semiconductor, as the LSPs present in the CdO NPs are located as high as 3,000 cm^−1^ (or 3.33 μm). Our NPs sustain broadband plasmons than can be exploited to enhance several molecular bond vibrations of the same molecule to fully fingerprint it, avoiding the risk of spectral detuning that is recurrent in this field [59]. All in all, we provide a novel framework that stems away from the current trends in SEIRA sensing. While most recent publications are highly focused on the detection with single nanostructures that rely on the use of a microscope, we apply SEIRA as a technique that can also excel at the centimeter scale. Our self-assembly process is capable of rapidly producing large areas (within the range of squared centimeters) of ready-to-sense surfaces that sustain vast numbers of nanostructures, yielding signal enhancements that scale linearly with surface coverage. Thus, our approach has the potential to yield high output, large area sensors, which could be readily exploited in commercial sensing applications.

## 4 Methods

### 4.1 Mid-IR spectroscopy

Infrared spectra were taken using a Fourier Transform Infrared (FTIR) spectrometer equipped with a deuterated triglycine sulfate (DTGS) detector, a KBr beamsplitter, and a SiC glowbar. The samples were measured in a transmittance configuration with an incident angle of 45° and p-polarized light (magnetic field perpendicular to the light incidence plane). All spectra were recorded with a resolution of 4 cm^−1^ and 1,024 iterations.

### 4.2 Nanoparticle growth and structure

The Cd(Zn)O NPs samples featured in this work were grown by MOCVD on doubled-side polished semi-insulating GaAs wafers, using tertiary butanol, dimethylcadmium, and diethylzinc as precursors, with N_2_ as the carrier gas. The nominal Zn molar concentration was varied between 0 and 20 %, and the temperature was maintained within the mass-transport-limited regime (300–350 °C) for the growth of NPs of the ternary compound Cd(Zn)O [54]. In contrast, for the binary oxide CdO NPs, the so-called “thermodynamically dominated” regime was used to prevent nanoparticle coalescence. The surface density and morphology of the samples were controlled by adjusting the growth conditions through several key parameters, including temperature, precursor flow rates, deposition time, and the sample position within the reactor, which proved to be particularly relevant due to the horizontal reactor geometry. These studies enabled the reproducible fabrication of samples with a wide range of particle densities and sizes while maintaining the hemispherical shape of the NPs. The samples’ characteristics and growth parameters are summarized in Table S1.

The structural characterization of the samples was done using a FEI Inspect F50 Scanning Electron Microscope (SEM) and a Dimension Icon Atomic Force Microscope (AFM) from Bruker. To evaluate the surface coverage of the samples, the SEM micrographs were analyzed using the particle analyzer tool from ImageJ free software. AFM scans were visualized and studied with the Gwyddion free software.

The samples are not intentionally doped. The high electron concentration in Cd(Zn)O is believed to originate from intrinsic defects that act as shallow donors, most likely hydrogen interstitials [60], [61], [62] whose concentration may be increased with increasing Zn content as a result of the gas precursors used for Zn [29].

### 4.3 SEIRA sensing

After the preparation and characterization of the nanoparticles, the samples were cleaned in two subsequent ultrasonic baths of acetone and isopropyl alcohol (IPA) for 10 min each. Then two different protocols were followed to deposit the PMMA layers depending on the desired thickness. For the 50 nm-thick layers, PMMA was directly spin-coated on the samples at 4,000 rpm for 60 s. For the 15 nm-thick layers, a solution of 10 % in volume of PMMA in acetone was spin-coated with the same parameters as the former case, followed by a thermal treatment on a hot plate at 160 °C for 2 min to evaporate any traces of the solvent. For both cases, an Eppendorf Research plus mechanical micropipette was used to drop cast 20 µL of the solution on the surface of the samples. The aforementioned thickness of the PMMA layers was calibrated by AFM on a bare GaAs substrate.

Once the samples with NPs are coated, they are measured in the spectrometer along with a GaAs substrate with a similar PMMA layer to act as a reference. The measured spectra are fitted using the smoothing algorithm proposed by Eilers [63]. The fit serves as a reference signal to standardize the measured data. Consequently, a predominantly flat line, except for the vibrational signals, is generated, enabling us to derive the vibrational signal intensity from this baseline-corrected spectrum by measuring the peak-to-peak amplitude of the vibrational signals [42].

For the measurement of SEIRA in vanillin, an alternate approach was carried out when depositing the compound. First, a 2 mg/mL solution of vanillin/IPA was prepared to then drop cast a 1 μL droplet of it onto the sample surface. The sample is left to dry for a few seconds and then measured in a Hyperion Vertex 70 FTIR microscope fitted with a × 36 Schwarzschild objective.

### 4.4 Finite elements method model

A 3D finite element model was created in COMSOL Multiphysics to analyze the electric field distribution in and around the NPs and PMMA layer. The model is comprised from top to bottom of the subsequent thin layers: Air, PMMA, and GaAs. The nanoparticles are sitting on the GaAs substrate either embedded on the PMMA layer or half-covered by it depending on the thickness of the layer. Conformal layers of PMMA were not considered for the sake of simplicity of the study. The chosen geometrical parameters were adapted from the AFM and SEM results obtained on the NPs samples. The following assumptions were made. The NPs are perfectly hemispherical and evenly distributed on the surface in an infinitely periodic lattice. Their radius is 30 nm and they are separated 60 nm from each other, thus complying to a mean surface coverage of 30 %. The light intensity enhancement maps shown on this work are calculated in the following manner. The values of |E_NP_|^2^ are obtained from the model with the NP and the values of |E_0_|^2^ from a simulation without it. When both field distributions are compared, the enhancement is obtained. The model uses linearly polarized light to emulate the incident beam of the FTIR spectrometer. The electric field is polarized in TM configuration and has an incidence angle of 45°.

## Supplementary Material

Supplementary Material Details
