# Supplementary material for: Large-area sensors using Cd(Zn)O plasmonic nanoparticles for surface-enhanced infrared absorption
Source: Nanophotonics. 2025 May 26;14(13):2237–49. doi: 10.1515/nanoph-2025-0020 (PMC12199560; doi:10.1515/nanoph-2025-0020)
Supplement: Supplementary file 1 — Supplementary Material Details [file j_nanoph-2025-0020_suppl_001.pdf]

Supporting information

Large-area sensors using Cd(Zn)O plasmonic nanoparticles for surface enhanced infrared absorption

P. Ibañez-Romero,[1] E. Martínez Castellano,[1] Javier Yeste,[2] F. Gonzalez-Posada,[3] T. Taliercio,[3] V. Muñoz-Sanjosé,[2] M. Montes Bajo,[1] and A. Hierro,[1]

[1] *ISOM, Universidad Politécnica de Madrid, Madrid, Spain*

[2] *Departament de Física Aplicada i Electromagnetisme, Universitat de València, Burjassot, Spain*

[3] *Univ. Montpellier, IES. UMR 5214, F-34000, Montpellier, France*

Sample directory

Table S1. A directory of the samples used in the study.

Sample name	Zn content (%)	Growth time (s)	Relative position in the reactor
A1	0	80	Front
A2	10	80	Front
A3	20	80	Front
B1	10	80	Front
B2	10	80	Back
B3	10	60	Front
B4	10	60	Back

S1. Surface characteristics dependance on Zn content

Figure S1 shows the surfaces for the three samples in series A, with different distinct Zn nominal contents. Sample A1 (0% Zn) shows a striking difference with the other two, being the NP's much smaller, similar in size to each other, and closer together. There is no apparent difference between the samples A2 (10% Zn) and A3 (20% Zn), which demonstrates that the deterioration of the plasmonic response of the latter is related to the loss mechanism presented by Tamayo et al [1].

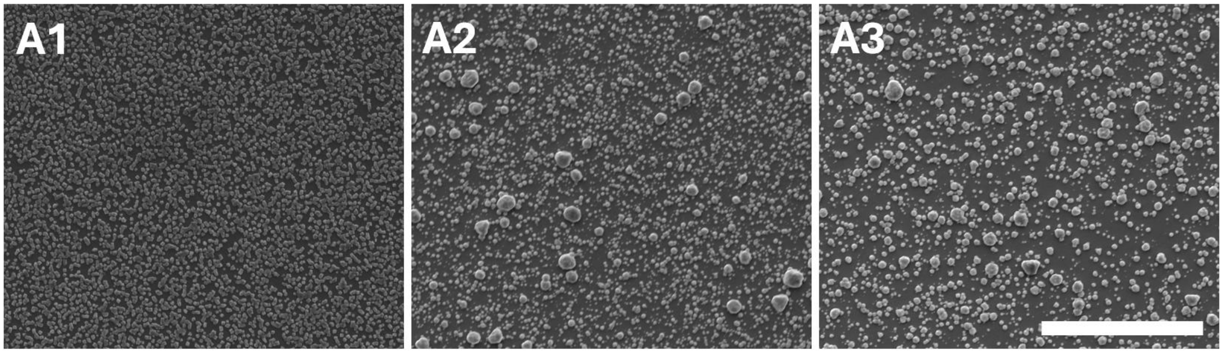

Figure S1. SEM micrographs of NPs with increasing Zn concentration: A1 (0% Zn), A2 (10% Zn) and A3 (20% Zn). The white bar measures 5 μm.

S2. Effects of interparticle distance.

As observed in Figure S1(A1), for high surface coverages, the NPs start to coalesce and transforms the system from being described as isolated NPs to behaving as a thin layer. This process is modeled in Figure S2.1, where the NPs are positioned at the nodes of a two-dimensional square

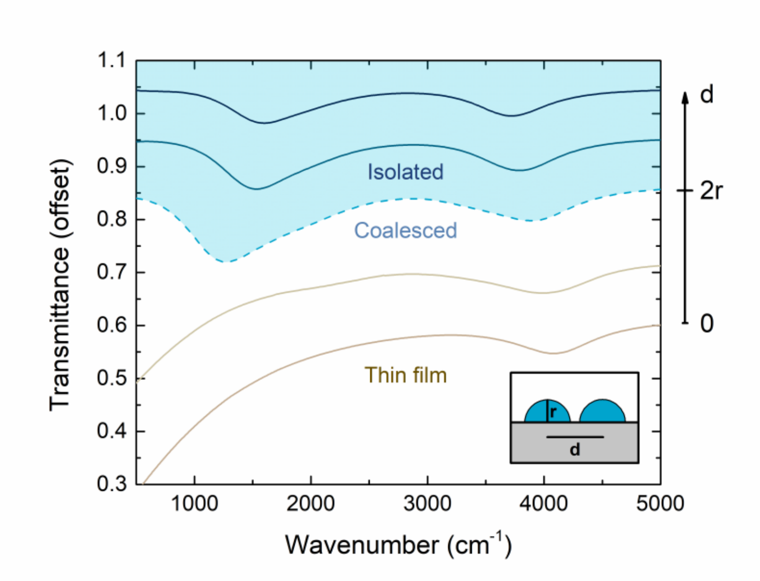

Figure S2.1: Effect of near field interaction and coalescence in a periodic model. The curves in the blue light field represent an uncoalesced stage with center-to-center distances (d in the figure) of 150 and 180 nm, larger than twice the radius of the NPs, $r = 30$ nm. Gray curves correspond to a coalesced stage with d values ranging from 120 (dashed line) to 60 nm (thin film limit) with an intermediate value of d = 100 nm.

periodic lattice. As the interparticle distance decreases, the system's transmittance progressively aligns with that of a continuous thin film.

The impact of the interparticle distance should also be studied in terms of their potential plasmonic coupling. Figure S2.2 presents the transmittance spectra for an array of identical, regularly spaced NPs, with nearest neighbor distances of 65 nm and 120 nm. The solid lines are obtained with the plasma broadening set to the real value, which yields two wide plasmonic modes at around 1700 cm^{-1} (low energy mode) and 3600 cm^{-1} (high energy mode). When the NPs are brought closer together, the high energy mode appears to shift to lower energies and the low energy mode to higher energies. This effect can be explained if the simulation is carried out at a (fictional) lower broadening, which is plotted in Figure S5 in dotted lines. The LE and HE modes are the convolution of smaller, sharper plasmonic resonances. In fact, when the NPs are brought together, new resonances appear as a result of the coupling between them. However, such effect is masked due to the intrinsic homogeneous broadening given by the nature of the NP's material.

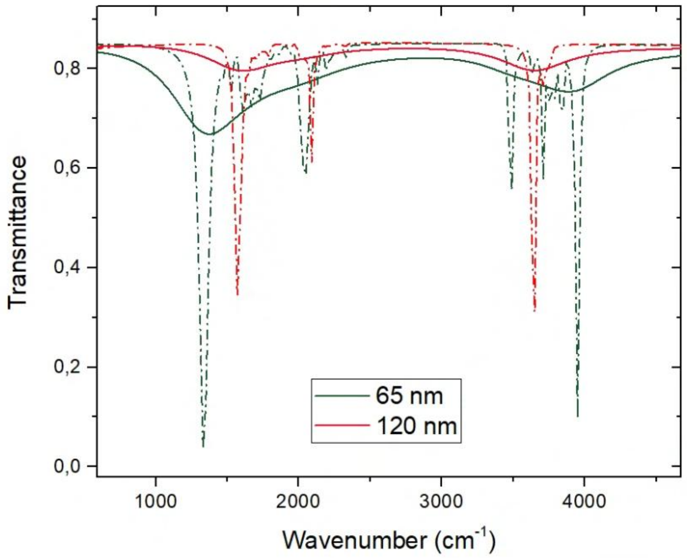

Figure S2.2. FEM transmittance simulations for an array of NPs with 30 nm radius separated (center to center) 65 nm (green) and 120 nm (red). The solid lines correspond to simulations carried out with the

actual plasma broadening (550 cm^{-1}) of the samples and the dotted lines to simulations with a very low, test broadening (10 cm^{-1}).

At the same time, inhomogeneous broadening happens due to the distribution of NP sizes and positions. The transmittance spectra shown in this work are obtained using a FTIR spectrometer which measures areas much bigger than the size of the NPs. Therefore, each measurement samples the signal of a large number of NPs with different sizes and interparticle distances. In fact, this is the reason why the LSPs from experimentally measured samples (800 – 1000 cm^{-1}) are broader than their numerical counterpart (550 cm^{-1}).

Finally, interparticle distance can also be studied in terms of the SEIRA vibrational signal. When the distance between the NPs is smaller than their radius, the low energy mode disappears and the high energy mode experiences a shift to higher energies, finally becoming the plasmonic resonance of a thin film (Figure S2.1). In terms of SEIRA, such changes translate into the effects shown in Figure S2.3, where the vibrational signals of a sample with fully coalesced NPs are shown. Due to the disappearance of the low energy mode, SEIRA enhancement also fades away. This can be seen in Figure S2.3 a and c, where the vibrational signal at the C=O bond absorption is equal when measured on the coalesced film and on the reference (naked GaAs). The underlying reason is that the coalescence of the particles into a thin film physically eliminates the hotspots necessary for the enhancement to happen. This is true for both the 15 nm and the 50 nm PMMA films. However, the case for the C-H bond absorptions coupled to the high energy LSP is different, as they are spectrally located close to the plasma frequency of the coalesced film (former high energy mode). As can be seen on Figures S2.3b and S2.3d, there is a higher vibrational signal for the case of the coalesced film than that of the GaAs reference. The enhancement is similar to what can be found in the case

of the isolated NPs. The high-energy mode of the LSP is related to out-of-plane oscillations of charge. Contrary to the low energy mode, the top and bottom interfaces of Cd(Zn)O do not disappear upon coalescence of the particles, therefore the SEIRA signal remains.

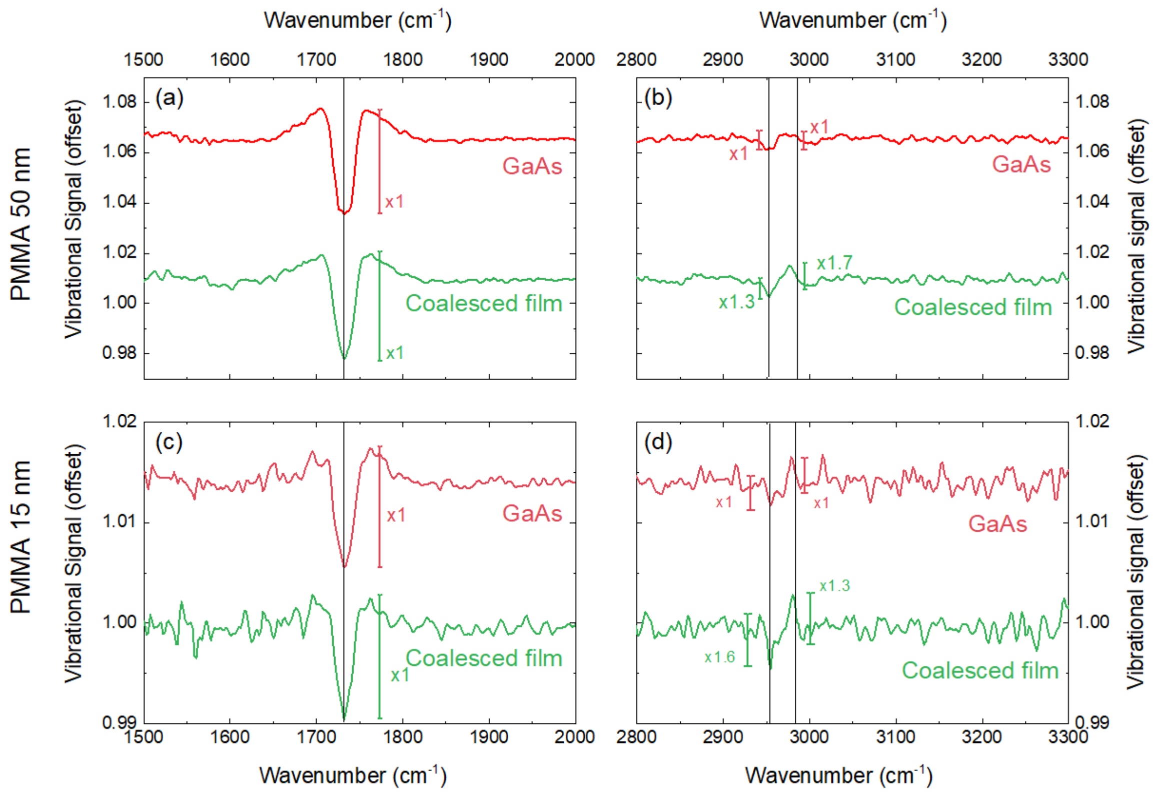

Figure S2.3. Vibrational signal of coalesced nanoparticles spin coated with a 50 nm (top) and a 15 nm (bottom) PMMA layer. The signals are obtained upon the subtraction from the spectra of a baseline obtained through Eilers' method [2]. The brackets indicate for each graph the strength of the bond absorptions using the GaAs measurement as reference.

S3. NP radius influence on plasmonic response

The position of the LSPs depends mostly on the plasma frequency of the NP's material, which is the same as that from high quality $Cd_{0.9}Zn_{0.1}O$ thin films on sapphire [3]. Other parameters as the NP radius have a small impact in the frequency of the resonances. Indeed, numerical models

(Figure S3.1) show that the low energy mode presents an almost unnoticeable redshift for larger radius (30 cm^{-1}), while the high energy mode shows a larger, yet still small shift (70 cm^{-1}). When such shifts are compared to the width of the resonances, which are around 600-900 cm^{-1}, they become negligible. These results allow us to confirm that despite the distribution of NP's sizes, the plasmonic response of all of them is similar in terms of the resonance energy.

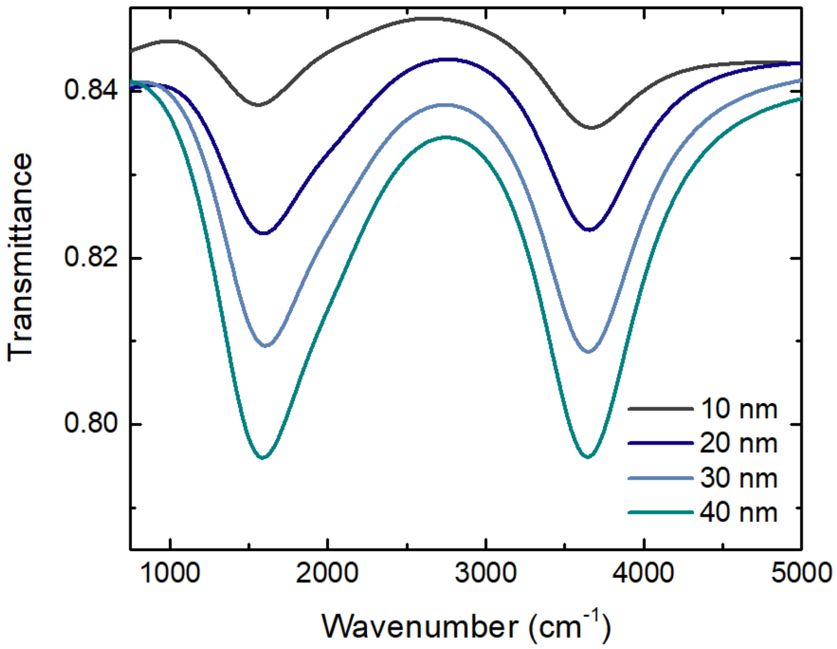

Figure S3.1 Simulated transmittance spectra of NPs with different radii, keeping the surface coverage constant, at 45° incidence.

In terms of the near field distribution, different NP sizes were analyzed. They are presented in Figure S3.2, and when compared to Figures 4c and 4d from the main manuscript it is clear that both the intensity of the effect and the spatial distribution of the field enhancement is almost identical for NPs with radius within the expected range in our samples.

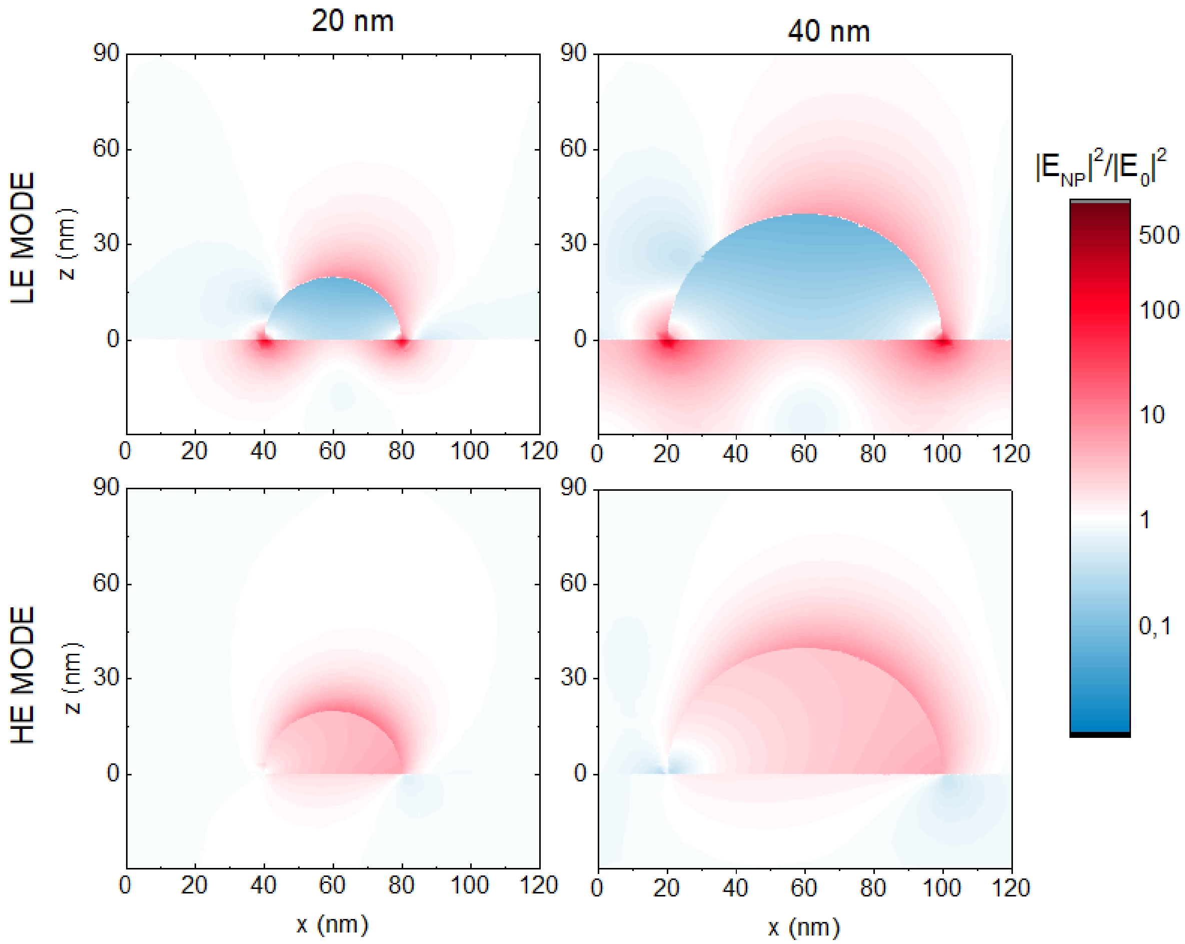

Figure S3.2. Normalized field enhancement for the low and high energy LSP modes at the cross section of NPs at Y = 60 nm with radius of 20 nm and 40 nm, at 1590 and 3640 cm^{-1}, respectively. The angle of incidence is 45º in all cases. The white color represents the areas where there is no enhancement when compared to a model without the NP.

S4. $Cd_{1-x}Zn_xO$ NP's tunability and model.

An FEM model of the NPs was created in Comsol. Figure S4 shows the transmitance spectra obtained experimentally (a) and through the model (b). The numerical results reproduce well the measurements in terms of how the resonance is shifted. However, in terms of the broadening and symmetry of the peaks, the agreement is not perfect, which is due to the self-assembled nature of the samples. Thus, the model is just a statistical approximation of the real sample's surface, and doesn't take into consideration inhomogeneities, shape of the NP size distribution (as shown in the histograms of Figure 2), or partial coalescence that takes place for instance in the CdO sample (with no Zn). In any case, as the plasmonic peaks are already significantly broader than the molecular absorption lines, the functionality of the NPs for SEIRA is not affected by a slight broadening of the modes.

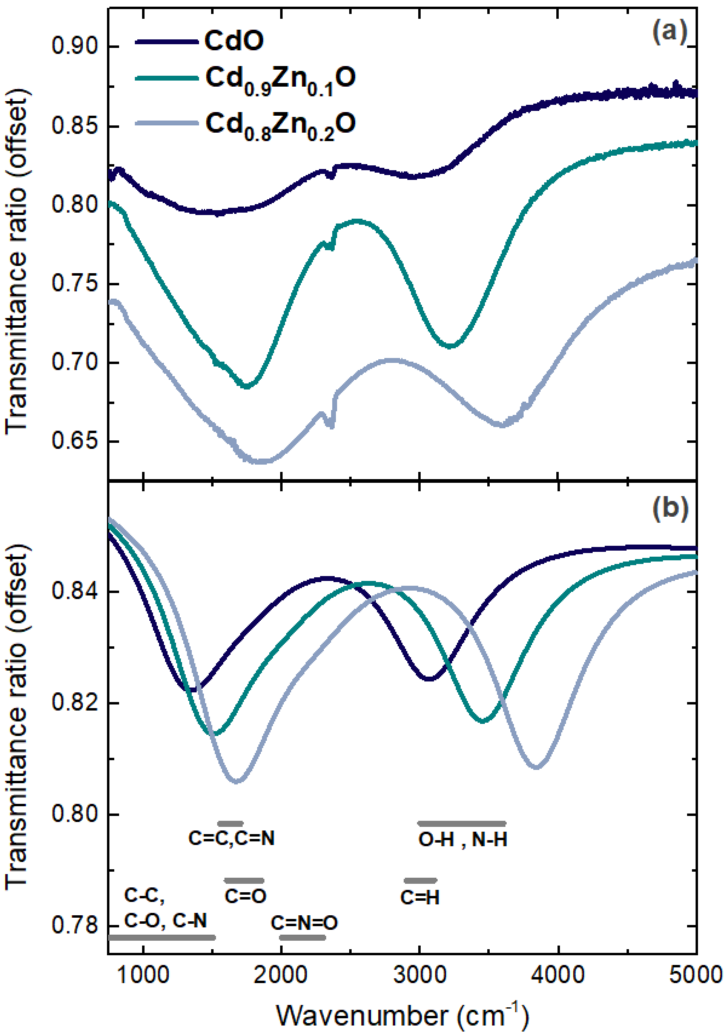

Figure S4.1. (a) Measured transmittance spectra of three Cd(Zn)O NP samples with different Zn alloy compositions at 45° incidence. (b) Simulated transmittance for three different Zn concentrations of 30 nm NPs at 45° incidence. The most significant absorption bands from molecular bonds are also shown.

The quality of the plasmonic response for different Zn content of the $Cd_{1-x}Zn_xO$ NP's is plotted in Figure S4.2. The figure of merit (FOM) is measured taking the ratio of the LSP frequency to the width of the mode, for both low and high energy modes for and the three Zn contents.

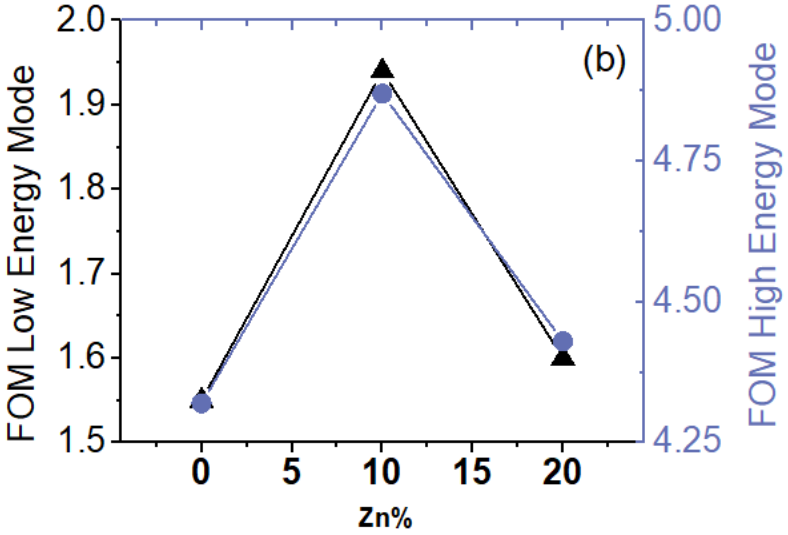

Figure S4.2 Figures of merit for both low and high energy modes for and the three Zn contents.

S5. SEIRA angle dependence.

The behavior of the low energy (LE) and high energy (HE) modes with varying angles of incidence is studied in detail in our earlier paper from 2021 [3]. For the low energy mode, due to the oscillation of the electric field in plane, the smaller the incidence angle (i.e. the larger the in-plane component of the electric field) the stronger the mode. This translates into a larger SEIRA signal for smaller angles of incidence, as shown in the experimental results of Figure S5. Conversely, the high energy mode is more intense as the angle of incidence is increased and this is reflected in the experimental results of Figure S5, where the SEIRA vibrational signal for the C=H PMMA bond is larger for larger angles of incidence.

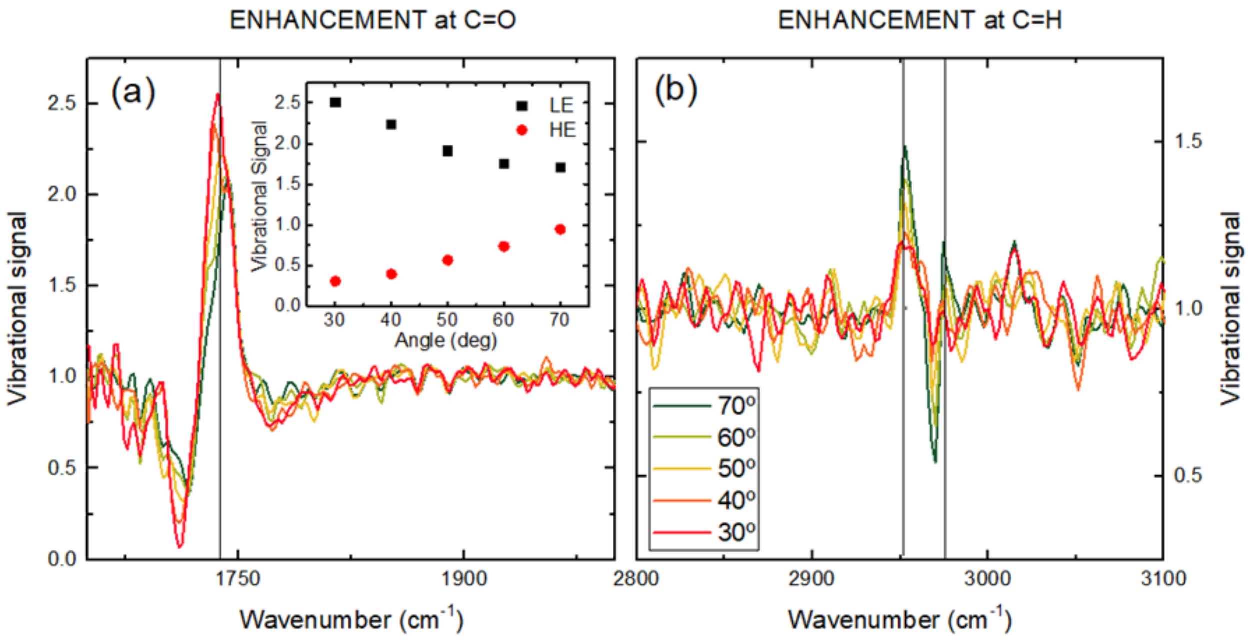

Figure S5. Vibrational signal measurements for varying angles of incidence on $Cd_{0.9}Zn_{0.1}O$ NPs. The solid black vertical lines mark the vibrational absorptions from the C=O bond (a) and the C-H bond (b) of PMMA. The insert in (a) shows the dependency of the vibrational signal with the angle of the low energy (LE) and high energy (HE) modes.

REFERENCES

[1] J. Tamayo-Arriola, A. Huerta-Barberà, M. Montes Bajo, E. Muñoz, V. Muñoz-Sanjosé, and A. Hierro, "Rock-salt CdZnO as a transparent conductive oxide," *Applied Physics Letters*, vol. 113, no. 22, p. 222101, Nov. 2018, doi: 10.1063/1.5048771.

[2] P. H. C. Eilers, "A Perfect Smoother," *Anal. Chem.*, vol. 75, no. 14, pp. 3631–3636, Jul. 2003, doi: 10.1021/ac034173t.

[3] E. Martínez Castellano *et al.*, "Self-assembled metal-oxide nanoparticles on GaAs: infrared absorption enabled by localized surface plasmons," *Nanophotonics*, vol. 10, no. 9, pp. 2509–2518, Jul. 2021, doi: 10.1515/nanoph-2021-0167.
